# Supplementary material for: Ecological Risk Assessment and Source Apportionment of Heavy Metals in the Soil of an Opencast Mine in Xinjiang
Source: Int J Environ Res Public Health. 2022 Nov 23;19(23):15522. doi: 10.3390/ijerph192315522 (PMC9736650; doi:10.3390/ijerph192315522)
Supplement: Supplementary file 1 [file ijerph-19-15522-s001.zip › ijerph-2026411-supplementary.pdf]

Supplementary Materials

Table S1. Classification of Modified Contamination Factor (MCF).

MCF classification	Contamination
MCF < 1.5	No pollution
1.5 ≤MCF < 2	Low pollution
2 ≤MCF < 4	Moderate pollution
4 ≤MCF < 8	High pollution
8 ≤MCF < 16	Higher pollution
16 ≤MCF < 32	Heavy pollution
MCF ≥ 32	Ultra-severe pollution

Table S2. Geoaccumulation index (I_{geo}) classification.

I_{geo}	Grade	Level of pollutant
0<	0	Almost no pollution
0-1	1	No pollution to moderate pollution
1-2	2	Moderate pollution
2-3	3	Moderate to heavy pollution
3-4	4	Heavy pollution
4-5	5	Heavy to seriously polluted
≥5	6	Seriously polluted

Table S3. Potential ecological risk assessment indicators and grading standards.

Risk grade	E_r^i	*RI*	Degree of ecological risk
Ⅰ	E_r^i<40	*RI*<150	Low
Ⅱ	40≤ E_r^i<80	150≤*RI*<300	Moderation
Ⅲ	80≤ E_r^i<160	300≤*RI*<600	High
Ⅳ	160≤ E_r^i<320	600≤*RI*<1200	Relatively high
Ⅴ	E_r^i ≫320	*RI*≫1200	Pole height

Table S4. Heavy metal toxicity response coefficient.

Heavy metal	Cd	Cu	Zn	Pb	As	Cr	Ni
Tr	30	5	1	5	10	2	5

Table S5. Parameter value of heavy metal exposure evaluation model.

Exposure hole parameter	Meaning / unit	Parameter value	
		child	adult
SA	Exposed skin area /(cm^2/d)	2800	5700
AF	Skin adhesion /(mg·cm^2/d)	0.2	1
ABS	Skin adsorption coefficient	0.001	0.001
EF	Exposed frequency /(d/a)	350	350
ED	Exposure duration /a	6	24
BW	Recipient weight /kg	29	63
AT (non-carcinogenic)	Average exposure time /d	365d×ED	365d×ED
AT (carcinogenic burden)	Average exposure time /d	365d×72a	365d×70a
PEF	Particulate emission factor (mg^3/kg)	1.36×10^9	1.36×10^9
IR_i	Respiratory inhalation volume /(m^3/d)	7.6	16
IR_s	Soil dust intake rate /(mg/d)	20	50

Table S6. RfD (mg/kg/day) and SF for seven heavy metals.

	Zn	Ni	Cu	Cr	Pb	As	Cd
RfD_{uptake}	3.00E-01	2.00E-02	4.00E-02	3.00E-03	3.50E-03	3.00E-04	1.00E-03
RfD_{skin}	6.00E-02	5.40E-03	1.20E-02	6.00E-05	5.25E-04	1.23E-04	1.00E-05
$RfD_{inhalation}$	3.00E-01	9.00E-05	1.43E-02	2.86E-05	3.50E-03	-	1.00E-05
SF_{uptake}	-	4.30E+01	-	5.00E-01	8.50E-03	1.50E+00	-
SF_{skin}	-	1.70E+00	-	-	-	3.66E+00	-
$SF_{inhalation}$	-	8.40E-01	-	4.20E+01	-	1.51E+01	6.30E+00
